# Supplementary material for: Effect of Pectin/Nanochitosan-Based Coatings and Storage Temperature on Shelf-Life Extension of “Elephant” Mango (Mangifera indica L.) Fruit
Source: Polymers (Basel). 2021 Oct 6;13(19):3430. doi: 10.3390/polym13193430 (PMC8512021; doi:10.3390/polym13193430)
Supplement: Supplementary file 1 [file polymers-13-03430-s001.zip › polymers-1337553-supplementary conv.pdf]

polymers

MDPI

Supplementary Materials: Effect of Pectin/Nanochitosan-Based Coatings and Storage Temperature on Shelf-life Extension of "Elephant" Mango (*Mangifera Indica* L.) Fruit

Thi Minh Phuong Ngo, Thanh Hoi Nguyen, Thi Mong Quyen Dang, Thi Van Thanh Do, Alissara Reungsang, Nareekan Chaiwong and Pornchai Rachtanapun

1. Effect of coating on shelf life and some quality traits of mango fruits

Table S1. Effect of coating on the weight loss of mango fruits.

Weight loss (%)						
**Storage time (day)**	**0**	**3**	**6**	**9**	**12**	**15**
**Uncoated**	0	6.9^{e} ± 0.8	16.4^{e} ± 1.2	21.8^{e} ± 1.3		
**0.75%NaCS**	0	5.6^{d} ± 0.3	8.9^{d} ± 0.4	12.9^{d} ± 0.5	16.5^{e} ± 0.7	
**1%NaCS**	0	4.5^{c} ± 0.2	7.2^{c} ± 0.3	10.2^{c} ± 0.4	13.8^{d} ± 0.5	
**1.25%NaCS**	0	3.3^{a} ± 0.2	6.0^{b} ± 0.3	9.0^{b} ± 0.4	12.8^{c} ± 0.5	15.8^{b} ± 0.8
**1.5%P/NaCS**	0	3.2^{a} ± 0.1	6.3^{b} ± 0.2	9.3^{bc} ± 0.5	12.2^{c} ± 0.6	
**2%P/NaCS**	0	2.2^{a} ± 0.2	4.1^{a} ± 0.1	7.1^{a} ± 0.3	9.6^{b} ± 0.4	12.5^{a} ± 0.9
**2.5%P/NaCS**	0	2.2^{a} ± 0.2	4.4^{a} ± 0.3	6.4^{a} ± 0.2	8.5^{a} ± 0.2	11.2^{a} ± 0.6

Note: Different lowercase letters (a, b, c) indicate significant differences between storage time (day) in the same columns (p≤0.05).

Table S2. Effect of coating on firmness of mango fruits.

Firmness (N)						
**Storage time (day)**	**0**	**3**	**6**	**9**	**12**	**15**
**Uncoated**	31.2^{aD} ± 1.5	14.2^{aC} ± 1.3	4.4^{aB} ± 0.9	0.9^{aA} ± 0.2		
**0.75%NaCS**	30.4^{aE} ± 1.3	18.6^{bD} ± 1.2	10.6^{bC} ± 0.9	5.3^{bB} ± 0.4	0.5^{aA} ± 0.2	
**1%NaCS**	31.8^{aE} ± 1.5	20.6^{bcD} ± 1.4	14.5^{cC} ± 1.1	7.6^{cB} ± 0.9	1.4^{aA} ± 0.5	
**1.25%NaCS**	31.2^{aF} ± 1.6	23.1^{dE} ± 1.3	19.5^{dD} ± 1.2	15.3^{dC} ± 1.0	6.8^{bB} ± 0.8	3.5^{bA} ± 0.4
**1.5%P/NaCS**	30.4^{aE} ± 1.3	22.5^{cdD} ± 1.2	16.5^{cC} ± 1.3	6.4^{bcB} ± 0.9	1.3^{aA} ± 0.1	
**2%P/NaCS**	31.8^{aF} ± 1.5	23.6^{deE} ± 1.7	18.7^{dD} ± 1.1	15.4^{dC} ± 0.7	7.5^{bB} ± 0.4	0.9^{aA} ± 0.4
**2.5%P/NaCS**	31.2^{aF} ± 1.6	25.8^{eE} ± 1.1	23.1^{eD} ± 1.4	19.9^{eC} ± 0.9	10.9^{cB} ± 0.6	7.0^{cA} ± 0.4

Note: Different lowercase letters (a, b, c) indicate significant differences between storage time (day) in the same columns (p≤0.05). Different uppercase letters (A, B, C) indicate significant differences between samples in the same rows (p≤0.05).

Table S3. Effect of coating on color (L*) of mango fruits.

L* of mango peel						
**Storage time (day)**	**0**	**3**	**6**	**9**	**12**	**15**
**Uncoated**	53.4^{aB} ± 3.1	54.8^{aB} ± 2.8	55.8^{aA} ± 4.1	72.1^{eA} ± 2.9		
**0.75%NaCS**	53.4^{aB} ± 3.3	53.9^{aB} ± 2.9	55.1^{aAB} ± 2.8	64.5^{dAB} ± 3.1	68.2^{cA} ± 3.1	
**1%NaCS**^{ns}	53.4^{aA} ± 2.9	52.6^{aA} ± 2.6	54.8^{aA} ± 2.8	59.5^{cA} ± 3.1	62.8^{dA} ± 3.3	
**1.25%NaCS**	53.4^{aAB} ± 2.7	52.9^{aA} ± 2.9	53.5^{aAB} ± 3.1	57.4^{bcAB} ± 3.2	59.9^{bcB} ± 3.4	63.4^{bA} ± 2.9
**1.5%P/NaCS**	53.4^{aA} ± 3.1	54.1^{aAB} ± 2.3	55.0^{aB} ± 2.6	58.7^{bcAB} ± 2.8	62.1^{cAB} ± 2.3	
**2%P/NaCS**	53.4^{aA} ± 3.1	55.2^{aAB} ± 2.6	54.2^{aAB} ± 2.7	53.5^{abBC} ± 2.3	56.7^{bC} ± 2.6	59.1^{bC} ± 3.0
**2.5%P/NaCS**	53.4^{aB} ± 3.1	53.1^{aB} ± 2.5	51.5^{aBC} ± 2.6	50.4^{aC} ± 2.4	47.6^{aB} ± 2.1	36.4^{aA} ± 2.3

Note: Different lowercase letters (a, b, c) indicate significant differences between storage time (day) in the same columns (p≤0.05). Different uppercase letters (A, B, C) indicate significant differences between samples in the same rows (p≤0.05).

Table S4. Effect of coating on color (a*) of mango fruits.

a* of mango peel						
**Storage time (day)**	**0**	**3**	**6**	**9**	**12**	**15**
**Uncoated**	-19.1^{aA} ± 3.1	-17.2^{aA} ± 2.8	-13.2^{aA} ± 4.1	2.3^{aB} ± 2.9		
**0.75%NaCS**	-19.1^{aA} ± 3.3	-17.5^{aA} ± 2.9	-14.1^{aA} ± 2.8	-5.2^{bB} ± 3.1	2.1^{bcC} ± 3.1	
**1%NaCS**	-19.1^{aA} ± 2.9	-18.1^{aA} ± 2.6	-15.1^{aA} ± 2.8	-6.3^{bC} ± 3.1	-0.1^{bD} ± 3.3	
**1.25%NaCS**	-19.1^{aA} ± 2.7	-18.2^{aA} ± 2.9	-15.9^{aA} ± 3.1	-7.4^{bB} ± 3.2	-0.8^{bB} ± 3.4	1.2^{bB} ±2.9
**1.5%P/NaCS**	-19.1^{aA} ± 3.1	-18.0^{aA} ± 2.3	-14.9^{aA} ± 2.6	-7.9^{bB} ± 2.8	5.7^{cC} ± 2.3	
**2%P/NaCS**	-19.1^{aA} ± 3.1	-18.4^{aA} ± 2.6	-17.1^{aA} ± 2.7	-16.2^{aA} ± 2.3	-8.3^{aB} ± 2.6	-6.7^{aB} ± 3.0
**2.5%P/NaCS**	-19.1^{aA} ± 3.1	-16.6^{aAB} ± 2.5	-12.3^{aB} ± 2.6	-3.4^{bC} ± 2.4	0.4^{bcCD} ± 2.1	2.4^{bD} ± 2.3

Note: Different lowercase letters (a, b, c) indicate significant differences between storage time (day) in the same columns (p≤0.05). Different uppercase letters (A, B, C) indicate significant differences between samples in the same rows (p≤0.05).

Table S5. Effect of coating on color (b*) of mango fruits.

b* of mango peel						
**Storage time (day)**	**0**	**3**	**6**	**9**	**12**	**15**
**Uncoated**	27.7^{aA} ± 3.1	39.7^{cB} ± 2.8	52.3^{dC} ± 4.1	33.3^{aA} ± 2.9		
**0.75%NaCS**	27.7^{aA} ± 3.3	35.2^{bcB} ± 2.9	53.4^{dC} ± 2.8	62.3^{dD} ± 3.1	51.3^{abC} ± 3.1	
**1%NaCS**	27.7^{aA} ± 2.9	32.2^{abA} ± 2.6	45.5^{cB} ± 2.8	52.3^{cC} ± 3.1	48.9^{abBC} ± 3.3	
**1.25%NaCS**	27.7^{aA} ± 2.7	31.2^{abAB} ± 2.9	39.5^{bABC} ± 3.1	47.2^{bC} ± 3.2	69.5^{cD} ± 18.8	45.6^{bBC} ± 2.9
**1.5%P/NaCS**	27.7^{aA} ± 3.1	29.1^{aAB} ± 2.3	32.5^{aAB} ± 2.6	34.1^{aB} ± 2.8	59.0^{bcC} ± 2.3	
**2%P/NaCS**	27.7^{aA} ± 3.1	28.8^{aA} ± 2.6	31.5^{aAB} ± 2.7	34.6^{aB} ± 2.4	46.4^{abC} ± 2.6	59.0^{cD} ± 3.0
**2.5%P/NaCS**	27.7^{aB} ± 3.1	30.5^{abBC} ± 2.5	35.0^{abCD} ± 2.6	35.2^{aD} ± 2.4	38.6^{aD} ± 2.1	13.3^{aA} ± 2.4

Note: Different lowercase letters (a, b, c) indicate significant differences between storage time (day) in the same columns (p≤0.05). Different uppercase letters (A, B, C) indicate significant differences between samples in the same rows (p≤0.05).

Table S6. Effect of coating on hue angle of mango fruits.

	**hue of mango peel**					
**Storage time (day)**	**0**	**3**	**6**	**9**	**12**	**15**
**Uncoated**	113.6^{a} ± 4.2	89.1^{a} ± 3.8	80.6^{a} ± 5.1	74.8^{a} ± 3.9		
**0.75%NaCS**	113.6^{a} ± 4.2	95.5^{ab} ± 3.9	87.2^{ab} ± 3.8	79.2^{ab} ± 4.1	63.4^{a} ± 4.1	
**1%NaCS**	113.6^{a} ± 4.2	101.5^{bc} ± 3.6	90.4^{bc} ± 3.9	79.9^{ab} ± 4.1	68.2^{a} ± 4.1	
**1.25%NaCS**	113.6^{aD} ± 4.2	108.6^{dD} ± 3.9	96.8^{cC} ± 4.1	85.6^{bcB} ± 4.2	79.9^{bAB} ± 4.4	74.1^{bA} ± 3.9
**1.5%P/NaCS**	113.6^{aD} ± 4.2	105.3^{cdC} ± 4.3	93.6^{bcB} ± 3.6	88.6^{cdB} ± 3.8	79.2^{bA} ± 4.3	
**2%P/NaCS**	113.6^{aC} ± 4.2	110.4^{dC}± 3.6	96.1^{cB} ± 3.8	94.0^{dAB} ± 4.3	90.9^{cAB} ± 3.6	86.7^{cA} ± 4.0
**2.5%P/NaCS**	113.6^{aE} ± 4.2	100.1^{bcD} ± 3.5	95.2^{cD} ± 3.6	80.3^{abC} ± 3.4	64.1^{aB} ± 4.1	56.8^{aA} ± 4.3

Note: Different lowercase letters (a, b, c) indicate significant differences between storage time (day) in the same columns (p≤0.05). Different uppercase letters (A, B, C) indicate significant differences between samples in the same rows (p≤0.05).

Table S7. Effect of coating on color (L*) of mango flesh.

	**L* of mango flesh**					
**Storage time (day)**	**0**	**3**	**6**	**9**	**12**	**15**
**Uncoated**	75.1^{aB} ± 3.5	77.0a^{B} ± 3.4	61.2^{aA} ± 2.8	59.9^{aA} ± 2.7		
**0.75%NaCS**	75.1^{aB} ± 3.5	76.8a^{B} ± 3.2	72.6^{bAB} ± 3.9	70.6^{bAB} ± 3.6	67.5^{aA} ± 3.5	
**1%NaCS**	76.8^{A} ± 3.4	76.2a^{A} ± 3.6	76.9^{bcA} ± 3.8	80.6^{cA} ± 3.7	76.9^{bA} ± 4.0	
**1.25%NaCS**	76.8a^{AB} ± 3.4	75.6a^{A} ± 3.9	77.9^{bcAB} ± 3.8	80.3^{cAB} ± 4.0	83.6^{bcB} ± 3.8	75.9^{bA} ± 3.2
**1.5%P/NaCS**	75.1a^{A} ± 3.5	77.5a^{AB} ± 3.4	83.4^{cB} ± 4.1	81.9^{cAB} ± 3.8	78.4^{bAB} ± 3.4	
**2%P/NaCS**	75.1a^{A} ± 3.5	76.4a^{AB} ± 3.4	76.9^{bcAB} ± 3.7	83.3^{cBC} ± 4.1	85.6^{cC} ± 4.2	88.9^{cC} ± 3.8
**2.5%P/NaCS**	75.1a^{B} ± 3.5	76.1a^{B} ± 2.0	79.9^{cBC} ± 3.8	83.4^{cC} ± 4.2	76.5^{bB} ± 3.5	58.2^{aA} ± 2.4

Note: Different lowercase letters (a, b, c) indicate significant differences between storage time (day) in the same columns (p≤0.05). Different uppercase letters (A, B, C) indicate significant differences between samples in the same rows (p≤0.05).

Table S8. Effect of coating on color (a*) of mango flesh.

	**a* of mango flesh**					
**Storage time (day)**	**0**	**3**	**6**	**9**	**12**	**15**
**Uncoated**	-19.2^{aA} ± 3.5	-8.5^{bB} ± 3.4	5.5^{cC} ± 2.8	2.7^{cC} ± 2.7		
**0.75%NaCS**	-19.2^{aA} ± 3.5	-11.8^{abB} ± 3.2	-7.2^{bBC} ± 3.9	-4.4^{bC} ± 3.6	-1.9^{bC} ± 3.5	
**1%NaCS**	-19.2^{aA} ± 3.4	-12.1^{abB} ± 3.6	-10.3^{abBC} ± 3.8	-4.3^{bCD} ± 3.7	-2.1^{bD} ± 4.0	
**1.25%NaCS**	-19.2^{aA} ± 3.4	-14.2^{abAB} ± 3.9	-12.5^{abAB} ± 3.8	-8.9^{abBC} ± 4.0	-5.2^{abC} ± 3.8	-3.2^{abC} ± 3.2
**1.5%P/NaCS**	-19.2^{aA} ± 3.5	-12.8^{abAB} ± 3.4	-8.9^{abB} ± 4.1	-5.7^{abB} ± 3.8		
**2%P/NaCS**	-19.2^{aA} ± 3.5	-16.8^{aAB} ± 3.4	-14.9^{aAB} ± 3.7	-11.7^{aBC} ± 4.1	-10.8^{aBC} ± 4.2	-6.2^{aC} ± 3.8
**2.5%P/NaCS**	-19.2^{aA} ± 3.5	-15.7^{aAB} ± 2.0	-9.9^{abBC} ± 3.8	-6.6^{abCD} ± 4.2	-2.7^{bDE} ± 3.5	1.6^{bE} ± 2.4

Note: Different lowercase letters (a, b, c) indicate significant differences between storage time (day) in the same columns ($p \leq 0.05$). Different uppercase letters (A, B, C) indicate significant differences between samples in the same rows ($p \leq 0.05$).

Table S9. Effect of coating on color (b*) of mango flesh.

	**b* of mango flesh**					
**Storage time (day)**	**0**	**3**	**6**	**9**	**12**	**15**
**Uncoated**	$62.1^{aC} \pm 3.5$	$58.6^{aBC} \pm 3.4$	$53.7^{aAB} \pm 2.8$	$48.3^{aA} \pm 2.7$		
**0.75%NaCS**	$62.1^{aD} \pm 3.5$	$59.0^{aCD} \pm 3.2$	$54.0^{aBC} \pm 3.9$	$49.1^{aAB} \pm 3.6$	$43.5^{bA} \pm 3.5$	
**1%NaCS**	$62.1^{aC} \pm 3.4$	$59.1^{aC} \pm 3.6$	$55.0^{aBC} \pm 4.8$	$49.9^{aAB} \pm 3.7$	$45.6^{bA} \pm 4.0$	
**1.25%NaCS**	$62.1^{aD} \pm 3.4$	$59.1a^{CD} \pm 3.9$	$54.1^{aBC} \pm 3.8$	$52.3^{aBC} \pm 4.0$	$47.3^{bA} \pm 3.8$	$40.3^{aA} \pm 3.2$
**1.5%P/NaCS**	$62.1^{aD} \pm 3.5$	$59.6^{aCD} \pm 3.4$	$55.1^{aBC} \pm 4.1$	$50.1^{aB} \pm 3.8$	$30.7^{aA} \pm 3.4$	
**2%P/NaCS**^{ns}	$62.1^{aA} \pm 3.5$	$62.9^{aA} \pm 3.4$	$63.2^{bA} \pm 3.7$	$64.1^{bA} \pm 4.1$	$66.6^{cA} \pm 4.2$	$69.0^{bA} \pm 3.8$
**2.5%P/NaCS**	$62.1^{aD} \pm 3.5$	$58.6^{aCD} \pm 2.0$	$53.4^{aC} \pm 3.8$	$45.9^{aB} \pm 4.2$	$41.2^{bAB} \pm 3.5$	$38.7^{aA} \pm 2.4$

Note: Different lowercase letters (a, b, c) indicate significant differences ($p \leq 0.05$). Different uppercase letters (A, B, C) indicate significant differences ($p \leq 0.05$).

Table S10. Effect of coating on hue angle of mango flesh.

	**hue of mango flesh**					
**Storage time (day)**	**0***	**3***	**6***	**9***	**12**	**15**
**Uncoated**	$99.7^{aB} \pm 4.8$	$95.8^{aAB} \pm 4.4$	$93.2^{aAB} \pm 3.9$	$90.1^{aA} \pm 4.0$		
**0.75%NaCS**^{ns}	$99.7^{aA} \pm 4.8$	$96.1^{a}A \pm 3.9$	$93.9^{aA} \pm 4.1$	$91.5^{aA} \pm 4.3$	$80.1^{aA} \pm 4.2$	
**1%NaCS**	$99.7^{aB} \pm 4.8$	$96.8^{aB} \pm 4.6$	$94.6^{aB} \pm 4.8$	$92.5^{aB} \pm 4.7$	$83.6^{abA} \pm 5.1$	
**1.25%NaCS**	$99.7^{aC} \pm 4.8$	$97.5^{aC} \pm 4.5$	$95.1^{aBC} \pm 4.8$	$93.1^{aBC} \pm 5.1$	$86.6^{abcAB} \pm 4.8$	$80.5^{aA} \pm 4.2$
**1.5%P/NaCS**	$99.7^{aB} \pm 4.8$	$98.4^{aAB} \pm 4.4$	$95.1^{aAB} \pm 5.1$	$92.1^{aAB} \pm 4.8$	$90.0^{bcA} \pm 4.4$	
**2%P/NaCS**^{ns}	$99.7^{aA} \pm 4.8$	$98.5^{aA} \pm 4.4$	$97.3^{aA} \pm 4.7$	$96.1^{aA} \pm 4.8$	$94.8^{cA} \pm 5.0$	$93.2^{bA} \pm 5.7$
**2.5%P/NaCS**	$99.7^{aC} \pm 4.8$	$97.3^{aC} \pm 3.0$	$95.0^{aBC} \pm 4.2$	$93.0^{aBC} \pm 4.8$	$87.5a^{bcB} \pm 4.5$	$75.7^{aA} \pm 4.4$

Note: Different lowercase letters (a, b, c) indicate significant differences ($p \leq 0.05$). Different uppercase letters (A, B, C) indicate significant differences ($p \leq 0.05$).

Table S11. Effect of coating on TSS of mango fruits.

	TSS (%)					
**Storage time (day)**	**0**	**3**	**6**	**9**	**12**	**15**
**Uncoated**	$4.7^{aA} \pm 0.2$	$13.0^{dB} \pm 0.6$	$17.1^{fC} \pm 0.7$	$15.7^{bD} \pm 0.6$		
**0.75%NaCS**	$4.7^{aA} \pm 0.2$	$9.5^{cB} \pm 0.6$	$16.5^{fC} \pm 0.7$	$20.6^{eD} \pm 0.9$	$19.4^{bD} \pm 0.9$	
**1%NaCS**	$4.7^{aA} \pm 0.2$	$9.1^{cB} \pm 0.4$	$15.3^{eD} \pm 0.6$	$18.9^{dE} \pm 0.7$	$22.1^{cF} \pm 0.9$	$20.4^{bC} \pm 1.0$
**1.25%NaCS**	$4.7^{aA} \pm 0.2$	$6.2^{bB} \pm 0.3$	$12.3^{cC} \pm 0.6$	$18.3^{cdD} \pm 0.8$	$20.9^{cE} \pm 0.9$	$19.5^{bE} \pm 0.9$
**1.5%P/NaCS**	$4.7^{aA} \pm 0.2$	$8.9^{cB} \pm 0.3$	$14.2^{dC} \pm 0.6$	$17.8^{cdD} \pm 0.9$	$22.4^{cE} \pm 1.0$	
**2%P/NaCS**	$4.7^{aA} \pm 0.2$	$5.6^{abA} \pm 0.2$	$10.2^{bB} \pm 0.5$	$17.1^{cC} \pm 0.6$	$18.2^{bD} \pm 0.7$	$20.1^{bE} \pm 0.9$
**2.5%P/NaCS**	$4.7^{aA} \pm 0.2$	$4.9^{aA} \pm 0.3$	$5.1^{aA} \pm 0.5$	$6.8^{aB} \pm 0.4$	$8.1^{aC} \pm 0.5$	$10.9^{aD} \pm 0.4$

Note: Different lowercase letters (a, b, c) indicate significant differences (p≤0.05). Different uppercase letters (A, B, C) indicate significant differences (p≤0.05).

Table S12. Effect of coating on TA of mango fruits.

	TA (%)					
**Storage time (day)**	**0**	**3**	**6**	**9**	**12**	**15**
**Uncoated**	$0.85^{aC} \pm 0.04$	$0.80^{aC} \pm 0.04$	$0.65^{aB} \pm 0.03$	$0.45^{aA} \pm 0.02$		
**0.75%NaCS**	$0.85^{aD} \pm 0.04$	$0.81^{aD} \pm 0.04$	$0.68^{abC} \pm 0.03$	$0.58^{bB} \pm 0.02$	$0.49^{aA} \pm 0.02$	
**1%NaCS**	$0.85^{aD} \pm 0.04$	$0.82^{aD} \pm 0.04$	$0.69^{abC} \pm 0.03$	$0.60^{bcB} \pm 0.03$	$0.55^{cB} \pm 0.02$	$0.48^{aA} \pm 0.03$
**1.25%NaCS**	$0.85^{aD} \pm 0.04$	$0.83^{aD} \pm 0.04$	$0.70^{bC} \pm 0.03$	$0.61^{bcB} \pm 0.02$	$0.58^{bB} \pm 0.02$	$0.52^{bA} \pm 0.03$
**1.5%P/NaCS**	$0.85^{aD} \pm 0.04$	$0.83^{aD} \pm 0.001$	$0.70^{bC} \pm 0.002$	$0.62^{bcB} \pm 0.001$	$0.52^{dA} \pm 0.001$	
**2%P/NaCS**	$0.85^{aC} \pm 0.04$	$0.83^{aC} \pm 0.04$	$0.75^{cB} \pm 0.03$	$0.70^{dB} \pm 0.03$	$0.60^{dA} \pm 0.03$	$0.56^{cA} \pm 0.02$
**2.5%P/NaCS**	$0.85^{aD} \pm 0.04$	$0.83^{aCD} \pm 0.002$	$0.81^{dC} \pm 0.001$	$0.77^{eB} \pm 0.002$	$0.74^{eB} \pm 0.001$	$0.70^{dA} \pm 0.001$

Note: Different lowercase letters (a, b, c) indicate significant differences (p≤0.05). Different uppercase letters (A, B, C) indicate significant differences (p≤0.05).

Table S13. Effect of coating on Vitamin C of mango fruits.

	Vitamin C (g/100 g fruit)					
**Storage time (day)**	**0**	**3**	**6**	**9**	**12**	**15**
**Uncoated**	$0.045^{aD} \pm 0.002$	$0.040^{aC} \pm 0.002$	$0.035^{aB} \pm 0.001$	$0.020^{aA} \pm 0.001$		
**0.75%NaCS**	$0.045^{aE} \pm 0.002$	$0.041^{abD} \pm 0.001$	$0.036^{aC} \pm 0.002$	$0.028^{bB} \pm 0.001$	$0.025^{aA} \pm 0.002$	
**1%NaCS**	$0.045^{aD} \pm 0.002$	$0.041^{abD} \pm 0.001$	$0.036^{aC} \pm 0.001$	$0.030^{bcB} \pm 0.002$	$0.027^{abAB} \pm 0.001$	$0.025^{aA} \pm 0.001$
**1.25%NaCS**	$0.045^{aD} \pm 0.002$	$0.042^{abC} \pm 0.002$	$0.037^{aB} \pm 0.001$	$0.032^{cdA} \pm 0.002$	$0.031^{abA} \pm 0.001$	$0.030^{bA} \pm 0.002$
**1.5%P/NaCS**	$0.045^{aE} \pm 0.002$	$0.041^{abD} \pm 0.001$	$0.037^{aC} \pm 0.002$	$0.033^{dB} \pm 0.001$	$0.029^{bcA} \pm 0.001$	
**2%P/NaCS**	$0.045^{aC} \pm 0.002$	$0.043^{bC} \pm 0.001$	$0.043^{bC} \pm 0.002$	$0.040^{eB} \pm 0.002$	$0.038^{dB} \pm 0.002$	$0.035^{cA} \pm 0.002$

**2.5%P/Na CS**	$0.045^{D} \pm 0.002$	$0.043^{bCD} \pm 0.002$	$0.042^{bCD} \pm 0.001$	$0.041^{eBC} \pm 0.002$	$0.039^{dB} \pm 0.001$	$0.036^{cA} \pm 0.001$

Note: Different lowercase letters (a, b, c) indicate significant differences (p≤0.05). Different uppercase letters (A, B, C) indicate significant differences (p≤0.05).

2. Effects of storage temperature on shelf life and some quality traits of mango fruits

Table S14. Effect of storage temperature on weight loss of mango fruits.

		**Weight loss (%)**								
**Temperature (°C)**	**coating**	**Storage time (days)**								
		**0**	**3**	**6**	**9**	**12**	**15**	**18**	**21**	**24**
17	uncoating	$6.9^{aB} \pm 0.8$	$5.4^{bA} \pm 0.2$	$9.3^{dC} \pm 0.4$	$15.8^{dD} \pm 0.7$	$21.7^{dE} \pm 0.9$	$26.7^{cF} \pm 1.2$			
	coating	$6.9^{aD} \pm 0.8$	$1.8^{aA} \pm 1.8$	$3.4^{aB} \pm 0.2$	$5.0^{aC} \pm 0.3$	$6.5^{aD} \pm 0.3$	$8.4^{aE} \pm 0.4$	$10.4^{F} \pm 0.5$	$12.8^{G} \pm 0.6$	$14.3^{H} \pm 0.6$
25	uncoating	$6.9^{aA} \pm 0.8$	$6.9^{cA} \pm 0.3$	$16.4^{eB} \pm 0.8$	$21.8^{eC} \pm 1.0$					
	coating	$6.9^{aC} \pm 0.8$	$2.2^{aA} \pm 0.1$	$5.2^{bB} \pm 0.3$	$8.5^{bD} \pm 0.4$	$11.2^{bE} \pm 0.5$	$15.8^{bF} \pm 0.7$			
32	uncoating	$6.9^{aA} \pm 0.8$	$7.5^{cA} \pm 0.3$	18.1^{fB} 0.9	$23.2^{fC} \pm 0.9$					
	coating	$6.9^{aB} \pm 0.8$	$3.0^{aA} \pm 0.1$	$7.7^{cB} \pm 0.3$	$12.6^{cC} \pm 0.6$	$17.0^{cD} \pm 0.8$				

Note: Different lowercase letters (a, b, c) indicate significant differences between storage time (day) in the same columns (p≤0.05). Different uppercase letters (A, B, C) indicate significant differences between temperature (°C) in the same rows (p≤0.05).

Table S15. Effect of storage temperature on firmness of mango fruits.

		**Firmness (N)**								
**Temperature (°C)**	**coating**	**Storage time (days)**								
		**0**	**3**	**6**	**9**	**12**	**15**	**18**	**21**	**24**
17	uncoating	$31.2^{aF} \pm 1.5$	$23.4^{cE} \pm 1.2$	$15.5^{dD} \pm 0.8$	$7.2b^{C} \pm 0.3$	$2.6^{aB} \pm 0.2$	$0.9^{aA} \pm 0.05$			
	coating	$31.8^{aI} \pm 1.5$	$27.6^{dH} \pm 1.3$	$23.4^{eG} \pm 1.2$	$19.9^{cF} \pm 0.9$	$15.4^{cE} \pm 0.7$	$10.3^{cD} \pm 0.5$	$6.1^{C} \pm 0.3$	$3.2^{B} \pm 0.2$	$1.2^{A} \pm 0.1$
25	uncoating	$31.2^{aD} \pm 1.50$	$13.1^{aC} \pm 0.6$	$8.4^{bB} \pm 0.4$	$0.9^{aA} \pm 0.01$					
	coating	$31.2^{aF} \pm 1.5$	$25.8^{dE} \pm 1.2$	$23.1^{eD} \pm 1.2$	$19.9^{cC} \pm 0.9$	$10.9^{bB} \pm 0.5$	$4.0^{bA} \pm 0.2$			
32	uncoating	$30.8^{aD} \pm 1.5$	$12.6^{aC} \pm 0.6$	$5.1^{aB} \pm 0.2$	$0.2^{aA} \pm 0.01$					
	coating	$30.4^{aE} \pm 1.5$	$20.5^{bD} \pm 1.0$	$13.8^{cC} \pm 0.6$	$6.6^{bB} \pm 0.3$	$2.5^{aA} \pm 0.1$				

Note: Different lowercase letters (a, b, c) indicate significant differences between storage time (day) in the same columns (p≤0.05). Different uppercase letters (A, B, C) indicate significant differences between temperature (°C) in the same rows (p≤0.05).

Table S16. Effect of storage temperature on color (L*) of mango fruits.

		**L* of mango fruits**								
**Temperature (°C)**	**coating**	**Storage time (days)**								
		**0**	**3**	**6**	**9**	**12**	**15**	**18**	**21**	**24**
17	uncoating	$53.4^{aAB} \pm 2.1$	$55.4^{abBC} \pm 1.9$	$57.2^{abBC} \pm 2.4$	$59.3^{bC} \pm 2.3$	$50.2^{aB} \pm 2.2$	$43.4^{aA} \pm 1.8$			
	coating	$53.4^{aA} \pm 2.1$	$53.4^{aA} \pm 2.6$	$53.7^{aA} \pm 2.5$	$53.9^{aAB} \pm 2.4$	$54.2^{abAB} \pm 2.3$	$54.8^{bAB} \pm 2.5$	$56.2^{AB} \pm 2.6$	$58.6^{BC} \pm 2.9$	$61.2^{C} \pm 2.5$
25	uncoating	$53.4^{aA} \pm 2.1$	$58.8^{bAB} \pm 2.3$	$55.8^{abB} \pm 2.4$	$72.1^{cC} \pm 2.8$					
	coating	$53.4^{aA} \pm 2.1$	$55.2^{abAB} \pm 2.6$	$54.2^{abAB} \pm 2.5$	$53.5^{aA} \pm 2.4$	$56.7^{bcBC} \pm 2.6$	$59.1^{bC} \pm 3.0$			
32	uncoating	$53.4^{aA} \pm 2.1$	$55.7^{abAB} \pm 2.1$	$58.9^{bB} \pm 2.6$	$68.8^{cC} \pm 2.9$					
	coating	$53.4^{aAB} \pm 2.1$	$52.1^{aA} \pm 2.4$	$54.6^{abAB} \pm 2.1$	$57.5^{abBC} \pm 2.3$	$59.8^{cC} \pm 2.9$				

Note: Different lowercase letters (a, b, c) indicate significant differences between storage time (day) in the same columns (p≤0.05). Different uppercase letters (A, B, C) indicate significant differences between temperature (°C) in the same rows (p≤0.05).

Table S17. Effect of storage temperature on color (L*) of mango flesh.

		**L* of mango flesh**								
**Temperature (°C)**	**coating**	**Storage time (days)**								
		**0**	**3**	**6**	**9**	**12**	**15**	**18**	**21**	**24**
17	uncoating	$75.1^{aA} \pm 3.2$	$77.5^{aAB} \pm 3.3$	$78.9^{aAB} \pm 3.1$	$82.7^{bB} \pm 3.9$	$82.7^{aB} \pm 2.5$	$82.8^{aB} \pm 3.8$			
	coating	$75.1^{aA} \pm 3.2$	$75.9^{aAB} \pm 3.6$	$77.1^{aABC} \pm 3.5$	$78.4^{abABC} \pm 3.9$	$81.1^{aABCD} \pm 3.8$	$85.9^{aD} \pm 3.8$	$83.1^{CD} \pm 3.8$	$82.4^{BCD} \pm 3.4$	$80.2^{ABCD} \pm 3.5$
25	uncoating	$75.1^{aA} \pm 3.2$	$77.0^{aA} \pm 2.6$	$81.2^{aA} \pm 3.1$	$79.9^{abA} \pm 3.2$					
	coating	$75.1^{aA} \pm 3.2$	$79.8^{aAB} \pm 3.1$	$76.9^{aA} \pm 3.5$	$83.3^{bB} \pm 2.6$	$83.3^{aB} \pm 3.4$	$83.3^{aB} \pm 3.6$			
32	uncoating	$75.1^{aA} \pm 3.2$	$78.2^{aA} \pm 3.1$	$80.4^{aA} \pm 2.8$	$75.0^{aA} \pm 2.6$					
	coating	$75.1^{aA} \pm 3.2$	$76.6^{aA} \pm 3.1$	$78.9^{aAB} \pm 3.0$	$80.8^{abAB} \pm 2.8$	$83.4^{aB} \pm 2.9$				

Note: Different lowercase letters (a, b, c) indicate significant differences between storage time (day) in the same columns (p≤0.05). Different uppercase letters (A, B, C) indicate significant differences between temperature (°C) in the same rows (p≤0.05).

Table S18. Effect of storage temperature on hue color of mango fruits.

		Hue color of mango fruits								
Temperature (°C)	coating	Storage time (days)								
		0	3	6	9	12	15	18	21	24
17	uncoating	113.6^{aD} ± 5.3	108.2^{bCD} ± 4.5	103.9^{cC} ± 4.3	95.7^{dB} ± 4.1	85.6^{abA} ± 3.9	79.5^{aA} ± 3.9			
	coating	113.6^{aG} ± 5.3	108.2^{bFG} ± 4.9	103.8^{cEF} ± 4.3	98.7^{dDE} ± 4.1	95.6^{cCD} ± 3.9	92.5^{bCD} ± 3.8	88.9^{BC} ± 3.8	84.5^{AB} ± 4.1	80.5^{A} ± 3.4
25	uncoating	113.6^{aC} ± 5.3	89.1^{aB} ± 4.1	86.1^{bB} ± 3.9	74.8^{bA} ± 3.5					
	coating	113.6^{aC} ± 5.3	110.3^{bC} ± 5.3	96.1^{cB} ± 4.5	94.0^{cdAB} ± 3.8	90.9^{cdAB} ± 4.3	86.8^{abA} ± 3.8			
32	uncoating	113.6^{aD} ± 5.3	89.2^{aC} ± 3.6	70.3^{aB} ± 2.9	52.1^{aA} ± 3.6					
	coating	113.6^{aC} ± 5.3	106.9^{bBC} ± 4.5	98.8^{cB} ± 4.5	87.5^{cA} ± 3.8	81.5^{aA} ± 3.9				

Note: Different lowercase letters (a, b, c) indicate significant differences between storage time (day) in the same columns (p≤0.05). Different uppercase letters (A, B, C) indicate significant differences between temperature (°C) in the same rows (p≤0.05).

Table S19. Effect of storage temperature on hue color of mango flesh.

		Hue color of mango flesh								
Temperature (°C)	coating	Storage time (days)								
		0	3	6	9	12	15	18	21	24
17	uncoating	99.7^{aB} ± 6.2	97.2^{abAB} ± 5.2	94.2^{bAB} ± 4.6	92.8^{bAB} ± 4.3	90.0^{aA} ± 4.6	88.7^{aA} ± 4.5			
	coating	99.7^{aD} ± 6.2	99.1^{bCD} ± 3.5	97.5^{bBCD} ± 3.8	96.2^{bBCD} ± 4.1	94.5^{aABCD} ± 2.9	93.2^{aABCD} ± 3.1	91.8^{ABC} ± 3.6	90.0^{AB} ± 4.1	88.4^{A} ± 4.2
25	uncoating	99.7^{aB} ± 6.2	96.7^{abAB} ± 4.2	93.2^{bAB} ± 4.1	90.1^{bA} ± 3.8					
	coating	99.7^{aA} ± 6.2	98.5^{bA} ± 4.5	97.3^{bA} ± 4.2	96.0^{bA} ± 4.1	94.8^{aA} ± 3.8	92.2^{aA} ± 3.5			
32	uncoating	99.7^{aD} ± 6.2	90.4^{aC} ± 3.5	80.4^{aB} ± 2.9	72.1^{aA} ± 3.6					
	coating	99.7^{aB} ± 6.2	96.3^{abAB} ± 3.8	94.1^{bAB} ± 3.8	92.1^{bAB} ± 4.1	89.5^{aA} ± 3.9				

Note: Different lowercase letters (a, b, c) indicate significant differences between storage time (day) in the same columns (p≤0.05). Different uppercase letters (A, B, C) indicate significant differences between temperature (°C) in the same rows (p≤0.05).

Table S20. Effect of storage temperature on the total microbial of mango fruits.

		log (CFU/g fruit)								
Temperature (°C)	coating	Storage time (days)								
		0	3	6	9	12	15	18	21	24
17	uncoating	1.2^{aA} ± 0.1	4.0^{cB} ± 0.2	6.3^{cC} ± 0.3	8.9^{cD} ± 0.4	10.9^{dE} ± 0.4	12.5^{cF} ± 0.5			
	coating	1.2^{aA} ± 0.1	1.7^{aB} ± 0.1	1.9^{aB} ± 0.1	2.3^{aC} ± 0.1	2.7^{aD} ± 0.1	3.6^{aE} ± 0.2	4.0^{F} ± 0.2	4.7^{G} ± 0.2	5.0^{H} ± 0.3
25	uncoating	1.2^{aA} ± 0.1	5.2^{dB} ± 0.3	8.9^{dC} ± 0.4	12.3^{dD} ± 0.4					
	coating	1.2^{aA} ± 0.1	2.7^{bB} ± 0.1	3.2^{bC} ± 0.2	4.7^{bD} ± 0.2	5.0^{bE} ± 0.3	5.3^{bE} ± 0.3			

		0	3	6	9	12	15	18	21	24
32	uncoating	1.2^{aA} ± 0.1	5.7^{eB} ± 0.3	10.0^{eC} ± 0.5	13.6^{eD} ± 0.6					
	coating	1.2^{aA} ± 0.1	2.4^{bB} ± 0.1	3.7^{bC} ± 0.2	4.8^{bD} ± 0.2	6.2^{cE} ± 0.3				

Note: Different lowercase letters (a, b, c) indicate significant differences between storage time (day) in the same columns (p≤0.05). Different uppercase letters (A, B, C) indicate significant differences between temperature (°C) in the same rows (p≤0.05).

Table S21. Effect of storage temperature on the count of yeast and mold of mango fruits.

		log (CFU/g fruit)								
**Temperature (°C)**	**coating**	**Storage time (days)**								
		**0**	**3**	**6**	**9**	**12**	**15**	**18**	**21**	**24**
17	uncoating	0.7^{aA} ± 0.1	1.6^{bB} ± 0.3	3.0^{dC} ± 0.2	4.9^{dD} ± 0.3	6.1^{dE} ± 0.4	7.4^{cF} ± 0.4			
	coating	0.7^{aA} ± 0.1	0.8^{aAB} ± 0.1	1.0^{aB} ± 0.1	1.5^{aC} ± 0.1	1.9^{aD} ± 0.2	2.3^{aE} ± 0.1	2.8^{F} ± 0.1	3.1^{G} ± 0.2	3.7^{H} ± 0.2
25	uncoating	0.7^{aA} ± 0.1	2.4^{cB} ± 0.1	5.9^{eC} ± 0.3	7.6^{eD} ± 0.6					
	coating	0.7^{aA} ± 0.1	1.0^{aB} ± 0.1	1.9^{bC} ± 0.1	2.4^{bD} ± 0.1	2.8^{bE} ± 0.1	3.6^{bF} ± 0.2			
32	uncoating	0.7^{aA} ± 0.1	3.9^{dB} ± 0.3	6.6^{fC} ± 0.4	8.6^{fD} ± 0.3					
	coating	0.7^{aA} ± 0.1	1.9^{bB} ± 0.3	2.5^{cC} ± 0.3	3.9^{cD} ± 0.2	4.6^{cE} ± 0.2				

Note: Different lowercase letters (a, b, c) indicate significant differences between storage time (day) in the same columns (p≤0.05). Different uppercase letters (A, B, C) indicate significant differences between temperature (°C) in the same rows (p≤0.05).

Table S22. Effect of storage temperature on TSS of mango fruits.

		TSS								
**Temperature (°C)**	**coating**	**Storage time (days)**								
		**0**	**3**	**6**	**9**	**12**	**15**	**18**	**21**	**24**
17	uncoating	4.7^{aA} ± 0.2	6.4^{bB} ± 0.3	9.1^{bC} ± 0.4	12.4^{bD} ± 0.3	17.5^{bE} ± 0.6	16.4^{bF} ± 0.4			
	coating	4.7^{aA} ± 0.2	5.1^{aA} ± 0.3	7.1^{a} ± 0.3	8.7^{aC} ± 0.6	10.3^{aD} ± 0.3	12.5^{aE} ± 0.3	15.6^{H} ± 0.4	17.9^{G} ± 0.5	19.5^{H} ± 0.6
25	uncoating	4.7^{aA} ± 0.2	13.0^{dB} ± 0.5	17.1^{dD} ± 0.5	15.7^{cC} ± 0.4					
	coating	4.7^{aA} ± 0.2	5.6^{abB} ± 0.2	10.2^{cC} ± 0.4	17.1^{dD} ± 0.5	18.2^{bE} ± 0.7	20.1^{cF} ± 0.7			
32	uncoating	4.7^{aA} ± 0.2	17.0^{eB} ± 0.8	19.5^{eC} ± 0.7	17.5^{dB} ± 0.7					
	coating	4.7^{aA} ± 0.2	7.2^{cB} ± 0.4	10.4^{cC} ± 0.4	14.8^{cD} ± 0.5	20.1^{cE} ± 0.7				

Note: Different lowercase letters (a, b, c) indicate significant differences between storage time (day) in the same columns (p≤0.05). Different uppercase letters (A, B, C) indicate significant differences between temperature (°C) in the same rows (p≤0.05).

Table S23. Effect of storage temperature on TA of mango fruits.

Temperature (°C)	coating	Storage time (days)								
		0	3	6	9	12	15	18	21	24
17	uncoating	0.85^{aD} ± 0.04	0.83^{bD} ± 0.04	0.70^{cC} ± 0.03	0.54^{cB} ± 0.02	0.50^{aB} ± 0.03	0.42^{aA} ± 0.02			
	coating	0.85^{aG} ± 0.04	0.84^{bG} ± 0.04	0.80^{eFG} ± 0.03	0.76^{eEF} ± 0.03	0.71^{cDE} ± 0.03	0.68^{cCD} ± 0.03	0.65^{BC} ± 0.03	0.61^{AB} ± 0.03	0.58^{A} ± 0.02
25	uncoating	0.85^{aC} ± 0.04	0.80^{bC} ± 0.04	0.65^{bB} ± 0.03	0.45^{bA} ± 0.02					
	coating	0.85^{aD} ± 0.04	0.83^{bD} ± 0.03	0.75^{dC} ± 0.03	0.70^{dB} ± 0.03	0.60^{bA} ± 0.02	0.56^{bA} ± 0.03			
32	uncoating	0.85^{aD} ± 0.04	0.63^{aC} ± 0.02	0.49^{aB} ± 0.02	0.38^{aA} ± 0.02					
	coating	0.85^{aC} ± 0.04	0.80^{bC} ± 0.03	0.72^{cdB} ± 0.03	0.67^{dB} ± 0.03	0.52^{aA} ± 0.02				

Note: Different lowercase letters (a, b, c) indicate significant differences between storage time (day) in the same columns (p≤0.05). Different uppercase letters (A, B, C) indicate significant differences between temperature (°C) in the same rows (p≤0.05).

Table S24. Effect of storage temperature on vitamin C of mango fruits.

Temperature (°C)	coating	vitamin C Storage time (days)								
		0	3	6	9	12	15	18	21	24
17	un-coating	0.045^{aE} ± 0.002	0.043^{cE} ± 0.002	0.039^{cD} ± 0.002	0.030^{cC} ± 0.001	0.018^{aB} ± 0.001	0.011^{aA} ± 0.001			
	coating	0.045^{aF} ± 0.002	0.044^{cF} ± 0.001	0.044^{dF} ± 0.003	0.042d^{EF} ± 0.003	0.040^{cDE} ± 0.003	0.037^{bCD} ± 0.002	0.035^{BC} ± 0.002	0.033^{AB} ± 0.001	0.030^{A} ± 0.002
25	un-coating	0.045^{aD} ± 0.002	0.040^{bC} ± 0.002	0.035^{bB} ± 0.001	0.020^{bA} ± 0.001					
	coating	0.045^{aD} ± 0.002	0.043^{cCD} ± 0.001	0.043^{dCD} ± 0.002	0.040^{dBC} ± 0.002	0.038^{cAB} ± 0.002	0.035^{bA} ± 0.002			
32	un-coating	0.045^{aD} ± 0.002	0.032^{aC} ± 0.001	0.015^{aB} ± 0.001	0.008^{aA} ± 0.002					
	coating	0.045^{aC} ± 0.002	0.040^{bC} ± 0.002	0.037^{bcC} ± 0.002	0.033^{cA} ± 0.002	0.030^{bA} ± 0.002				

Note: Different lowercase letters (a, b, c) indicate significant differences between storage time (day) in the same columns (p≤0.05). Different uppercase letters (A, B, C) indicate significant differences between temperature (°C) in the same rows (p≤0.05).
